# Limbitis Secondary to Autologous Serum Eye Drops in a Patient with Atopic Keratoconjunctivitis

**DOI:** 10.1155/2011/576521

**Published:** 2011-12-21

**Authors:** Jeffrey David Welder, Pejman Bakhtiari, Ali R. Djalilian

**Affiliations:** Illinois Eye and Ear Infirmary, University of Illinois at Chicago, 1855 West Taylor Street, Chicago, IL 60612, USA

## Abstract

*Purpose*. Report a case of limbitis secondary to autologous serum eye drops in a patient with atopic keratoconjunctivitis. *Design*. Interventional case report. *Methods*. A 32-year-old African American female with atopic keratoconjunctivitis (AKC) presented with chronic dry eye and diffuse punctate epithelial erosions refractory to conservative treatment. She was initially managed with cyclosporine ophthalmic 0.05% in addition to preservative-free artificial tears and olopatadine hydrochloride 0.2% for 6 months. She was later placed on autologous serum eye drops (ASEDs) and 4 weeks into treatment developed unilateral limbitis. The limbitis resolved shortly after stopping ASEDs in that eye; however, the drops were continued in the contralateral eye, which subsequently developed limbitis within 2 weeks. ASEDs were discontinued in both eyes, and the patient has remained quiet ever since. *Results*. Patient with a history of AKC and no prior history of limbitis developed limbitis shortly after starting ASEDs, which resolved promptly after discontinuation of therapy with no subsequent recurrence of inflammation. *Conclusion*. ASEDs are widely used in the treatment of complicated or treatment refractory dry eye. The potential side effects should be kept in mind when prescribing ASEDs for any patient, especially in those with underlying immunological diseases and circulating inflammatory factors.

## 1. Introduction

Ocular surface homeostasis is maintained by adequate tear production, a stable tear film, and healthy eyelid and ocular surface anatomy [1]. Aberrations in any of these components can lead to dry eye and secondary complications of epithelial erosions, ulceration, scarring, melting, or perforation [2]. Tears play an important role in ocular surface stability by lubricating the eye and preventing corneal desiccation as well as by providing nutritional, epitheliotropic, antimicrobial, and antiapoptotic factors to the cornea and conjunctiva [3].

The treatment of dry eye is based upon the inciting cause, the disease severity, and the presence of secondary complications. Most cases of mild-to-moderate dry eye can be managed with conventional therapy including preservative-free artificial tear substitution, anti-inflammatory medications such as cyclosporine ophthalmic, punctal occlusion, or lid revision [1]. However, in cases of severe dry eye or in the presence of secondary complications such as persistent epithelial defects or visual impairment, more intensive therapy is necessitated [2]. Autologous serum eye drops (ASEDs) have been recommended for use in these cases given their ability to both lubricate the eye and enhance the growth and migration of the corneal epithelium via the presence of nutritional, epitheliotropic, antiapoptotic, and antimicrobial factors similar to natural tears [3].

However, there are reported complications associated with the use of ASEDs [4]. In this paper, we describe the case of a patient with atopic keratoconjunctivitis (AKC) who was treated with ASEDs for dry eye associated with epithelial erosions and subsequently developed bilateral limbitis.

## 2. Case Report

A 32-year-old African-American female with a history of atopic keratoconjunctivitis (AKC) was referred to our clinic for chronic dry eye and diffuse punctate epithelial erosions refractory to conservative treatment including preservative-free artificial tears (Systane; Alcon, Fort Worth, Texas, USA), olopatadine hydrochloride 0.2% (Patanol; Alcon, Fort Worth, Texas, USA), and other topical mast cell stabilizers. On presentation, she complained of ocular discomfort and foreign body sensation that was worse upon waking and relieved only temporarily by artificial tears. She noted blurring of her vision over the previous two months but denied any symptoms of pain or red eye during the previous year. Review of systems was notable for a history of eczema. Her past ocular history was significant for AKC and bilateral open angle glaucoma. Her glaucoma was managed with bilateral tube shunts and was stable. Past medical history was unremarkable. She was taking loratadine daily for allergies but no other medications. Family and social history were noncontributory.

On examination, the patient's corrected visual acuity was 20/200 OD and 20/100 OS. IOP was 12 mmHg OD and 14 mmHg OS with tube shunts in place. External examination revealed bilateral moderate-to-severe dry eye with diffuse punctate epithelial erosions. Conjunctiva was clear OU. Decreased corneal sensation was present OU. Bilateral 1-2 plus cortical cataract with a clear optical zone was noted. Schirmer's testing (with anesthetic) revealed 4 mm of wetting OD and 5 mm OS.

A clinical diagnosis of AKC with dry eye refractory to conservative medical management was made, and the patient was started on cyclosporine ophthalmic 0.05% (Restasis; Allergan, Irvine, California) four times daily in addition to preservative-free artificial tears (Systane) and olopatadine hydrochloride 0.2% (Patanol) twice daily. The patient was followed closely over the next 6 months and failed to progress clinically. Follow-up exams had all shown diffuse epitheliopathy with clear conjunctiva bilaterally. At that point, ASEDs every two hours were added to the patient's regimen. The ASEDs were prepared by obtaining peripheral blood (40 mL) from the antecubital fossa and centrifuging at 1500 to 3000 for 20 minutes. A 20 percent serum dilution was prepared in a balanced saline solution and stored for use in a sterile bottle at −20°C. Each bottle was removed for use and kept at 4°C between applications.

Within a few weeks of starting treatment, the patient returned to clinic with a 1-week history of a red right eye. Her vision remained stable. Examination revealed moderate limbal injection and elevation in the right eye. Left eye was quiet. Corneal exam was unchanged. A diagnosis of limbitis OD was made. It was suspected that the patient could be having a reaction to the ASEDs given the prompt development of limbitis after starting the drops with no similar episodes observed during the previous several years of observation. ASEDs were discontinued OD with the initiation of prednisolone acetate ophthalmic 1.0% (Pred Forte; Allergan, Irvine, California) four times daily for inflammation management. Other topicals were continued OU, and ASEDs were continued OS.

**Figure 1:**
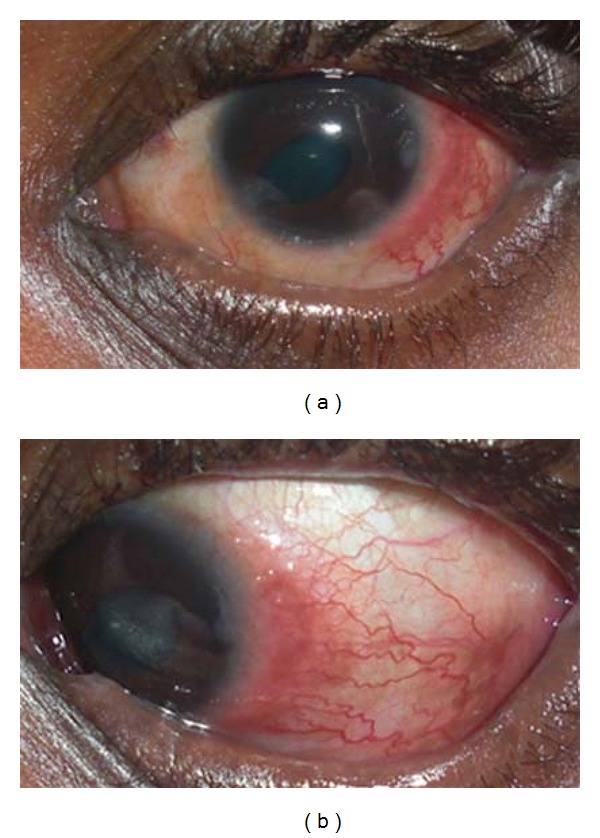
External photographs of the patient reported in our case. Marked temporal limbal injection and focal conjunctival elevation is apparent.

At the two-week followup, patient reported prompt resolution of the limbitis in the right eye within 2 days of stopping ASEDs, however, had developed interval limbitis OS with a presentation similar to the contralateral eye (Figure 1). At that time, the ASEDs were considered the most likely cause of the patient's reaction and were completely discontinued. Patient similarly had prompt resolution of the limbitis after stopping the ASEDs.

After 15 months of followup, the patient has shown no signs or symptoms of limbitis in either eye. Patient continues to experience symptomatic dry eye and diffuse corneal epitheliopathy despite aggressive therapy. She is currently being considered for a scleral contact lens.

## 3. Discussion

The use of ASEDs has been accepted as an alternative to artificial tears for the management of severe dry eye and its complications. The tear film, as described above, maintains ocular surface homeostasis through its nutritional as well as epitheliotropic, antimicrobial, and antiapoptotic abilities [3]. ASEDs, like natural tears, contain bioactive factors including vitamin A, epithelial growth factor (EGF), transforming growth factor-*β* (TGF-*β*), fibronectin, albumin, *α*-2 macroglobulin, the platelet-derived growth factor (PDGF-AB), hepatocyte growth factor, and neuropeptides [5, 6]. These factors promote the growth and migration of corneal epithelium allowing for more rapid healing of defects.

It has been shown that ASEDs are superior to artificial tears in the management of dry eye and have been demonstrated to effectively manage Sjogren-type dry eye [7], persistent epithelial defects, neurotrophic keratopathy [8], chronic graft-versus-host disease [9], superior limbic keratoconjunctivitis [10], and in LASIK-induced dry eye [11]. Yet, a number of complications are associated with ASEDs therapy. As they are derived from blood, consideration of anemia, systemic disease, and blood-borne infection is required [4]. ASEDs dropper-bottle microbial contamination has also been reported, requiring strict sterile technique and storage [12]. One group has described immunoglobulin deposition in the cornea after ASEDs therapy for persistent epithelial defect in recurrent herpes simplex keratitis [13]. In addition, ASEDs have been rarely associated with discomfort, epitheliopathy, bacterial conjunctivitis, and eye lid eczema [4]. Previous studies have used concentrations of ASEDs ranging from 20% to 100%, with no clear consensus on dose or concentration effect established [14].

In our patient with AKC, limbitis occurred after just few weeks of 20% ASEDs therapy in a patient that had been quiet for the previous several years of observation, making an adverse reaction the most likely cause of her inflammation. AKC is a chronic ocular surface allergic inflammatory condition that is sometimes refractory to conventional topical therapy [15]. Immunologic findings in atopy include increased levels of immunoglobulin E (IgE), eosinophils, spontaneous histamine release from mast cells, and Th2 cells secreting interleukin-4 (IL-4) and IL-5, and decreased Th1 cells secreting interferon *γ* [16]. While the exact cause of our patient's reaction remains unclear, it is possible that immunologically active ingredients in the serum of atopic patients could trigger ocular surface inflammation. These mediators may gain access to the ocular surface in higher concentrations with ASEDs, or these drops may allow those mediators to bypass the partially impaired barrier between the blood and ocular surface microenvironments.

This case provides evidence that ASEDs should be used with close followup, especially in patients with AKC or other immunological disorders. For example, we observed another patient with ocular cicatricial pemphigoid and keratinization whose ocular surface inflammation acutely worsened following treatment with ASEDs. In addition, it is possible that proinflammatory factors in the serum could exacerbate ocular surface conditions after topical therapy. In these situations, the use of ASEDs with topical anti-inflammatory therapy and/or close followup may be advised.
